# Updated global epidemiology atlas of human prion diseases

**DOI:** 10.3389/fpubh.2024.1411489

**Published:** 2024-06-13

**Authors:** Li-Ping Gao, Ting-Ting Tian, Kang Xiao, Cao Chen, Wei Zhou, Dong-Lin Liang, Run-Dong Cao, Qi Shi, Xiao-Ping Dong

**Affiliations:** ^1^National Key-Laboratory of Intelligent Tracking and Forecasting for Infectious Disease, National Health Commission Key Laboratory of Medical Virology and Viral Diseases, National Institute for Viral Disease Control and Prevention, Chinese Center for Disease Control and Prevention, Beijing, China; ^2^Center for Biosafety Mega-Science, Chinese Academy of Sciences, Wuhan, China; ^3^China Academy of Chinese Medical Sciences, Beijing, China; ^4^Shanghai Institute of Infectious Disease and Biosafety, Shanghai, China

**Keywords:** human prion disease, CJD, cases, mortality, surveillance

## Abstract

**Introduction:**

Human prion disease (PrD), a group of fatal and transmissible neurodegenerative diseases, consists of Creutzfeldt–Jakob disease (CJD), kuru, fatal familial insomnia (FFI), Gerstmann-Sträussler-Scheinker disease (GSS), and variably protease-sensitive prionopathy (VPSPr). The emergence of bovine spongiform encephalopathy (BSE) in cattle and variant CJD (vCJD) has greatly threatened public health, both in humans and animals. Since the 1990's, dozens of countries and territories have conducted PrD surveillance programs.

**Methods:**

In this study, the case numbers and alternative trends of different types of PrD globally and in various countries or territories from 1993 to 2020 were collected and analyzed based on the data from the websites of the international and national PrD surveillance programs, as well as from relevant publications.

**Results:**

The total numbers of the reported PrD and sporadic CJD (sCJD) cases in 34 countries with accessible annual case numbers were 27,872 and 24,623, respectively. The top seven countries in PrD cases were the USA (*n* = 5,156), France (*n* = 3,276), Germany (*n* = 3,212), Italy (*n* = 2,995), China (*n* = 2,662), the UK (*n* = 2,521), Spain (*n* = 1,657), and Canada (*n* = 1,311). The annual PrD case numbers and mortalities, either globally or in the countries, showed an increased trend in the past 27 years. Genetic PrD cases accounted for 10.83% of all reported PrD cases; however, the trend varied largely among the different countries and territories. There have been 485 iatrogenic CJD (iCJD) cases and 232 vCJD cases reported worldwide.

**Discussion:**

The majority of the countries with PrD surveillance programs were high- and upper-middle-income countries. However, most low- and lower-middle-income countries in the world did not conduct PrD surveillance or even report PrD cases, indicating that the number of human PrD cases worldwide is markedly undervalued. Active international PrD surveillance for both humans and animals is still vital to eliminate the threat of prion disease from a public health perspective.

## Introduction

Human prion diseases (PrDs) are a group of progressive, fatal neurodegenerative diseases caused by the post-translational misfolding of a normal host protein. These diseases are potentially transmissible, and the infective agent, the prion, is considered to consist principally or solely of the abnormally folded protein (1). The disease-associated prion protein (PrP^Sc^) shares the same amino acid sequence as the host normal cellular prion protein (PrP^C^). Human PrDs have sporadic, inherited, and acquired forms. The majority of human PrDs are in sporadic form, namely sporadic Creutzfeldt–Jakob disease (sCJD) (2, 3). Approximately 10–15% of the PrDs are inherited, which is associated with the various mutations in Prion Protein gene (*PRNP*)-encoding prion protein (PrP). Clinically, genetic prion diseases include genetic CJD (gCJD), fatal familial insomnia (FFI), and Gerstmann-Sträussler-Scheinker disease (GSS) (2, 4). Acquired PrDs include kuru, which is an endemic disease closely related to ritualistic mortuary cannibalism; iatrogenic CJD (iCJD), which is associated with different medical interventions; and variant CJD (vCJD), which is caused by consuming products contaminated with bovine spongiform encephalopathy (BSE) agents. In the recent decade, a new form of PrD, namely variably protease-sensitive prionopathy (VPSPr), has been documented. This form appears sporadically but is distinct from sCJD, both clinically and pathologically (3, 5, 6).

Despite the recognition of CJD in the early 1920's, broad awareness of CJD or PrD in public has occurred in the recent decades since the outbreak of BSE in cattle and the emergence of vCJD in humans (7, 8). Under the framework of the World Health Organization (WHO), a global surveillance program was implemented in the 1990's, consisting of many countries in Europe and North America and some countries in Oceania, Asia, and South America. Those national and international surveillance activities have remarkably improved the disease diagnosis, the research and development of new diagnostic tools, and public awareness. Even an International CJD Awareness Day (12 November) has been proposed to improve the awareness of civilians and raise concern within the whole society (9).

As a kind of rare neurological disease that is estimated at ~1–2 deaths per million population per year, human PrDs are still underdiagnosed in many countries. The epidemiology of sCJD is poorly understood outside of Europe, North America, Australia, and several countries in Asia. Meanwhile, the recent emergence of various animal PrDs, such as chronic wasting disease (CWD) outside North America (10–12) and prion disease in dromedary camels in Algeria (13), indicates potential threats to public health. In this study, we reviewed the general surveillance data of human PrDs in more than 30 countries from 1993 to 2020 and analyzed the correlation between the diseases and onset ages. It is apparent that the case numbers and the annual mortality rates (AMRs) of human PrDs have increased in the recent decades.

## Materials and methods

### Case definition, data source, and process

The definition of human PrDs was based on the diagnostic criteria for CJD issued by the WHO (14). All PrD or CJD cases here included those fulfilled with the criteria of probable and definite diagnosis. The relevant PrD data, that is, the annual case numbers of the different types of CJD and annual mortalities (per million) of the many European countries from 1993 to 2020, were obtained from the CJD International Surveillance Network EuroCJD (15). The data from other countries, such as the UK, Japan, Korea, China, the USA, and Canada, were obtained from other websites and literature studies (16–19). Microsoft Excel 2021, GraphPad Prism 5.0 (GraphPad Software Inc., San Diego, California), and the R language were used for data processing.

## Results

### Spatiotemporal distribution of all PrD and sCJD cases

**Figure 1:**
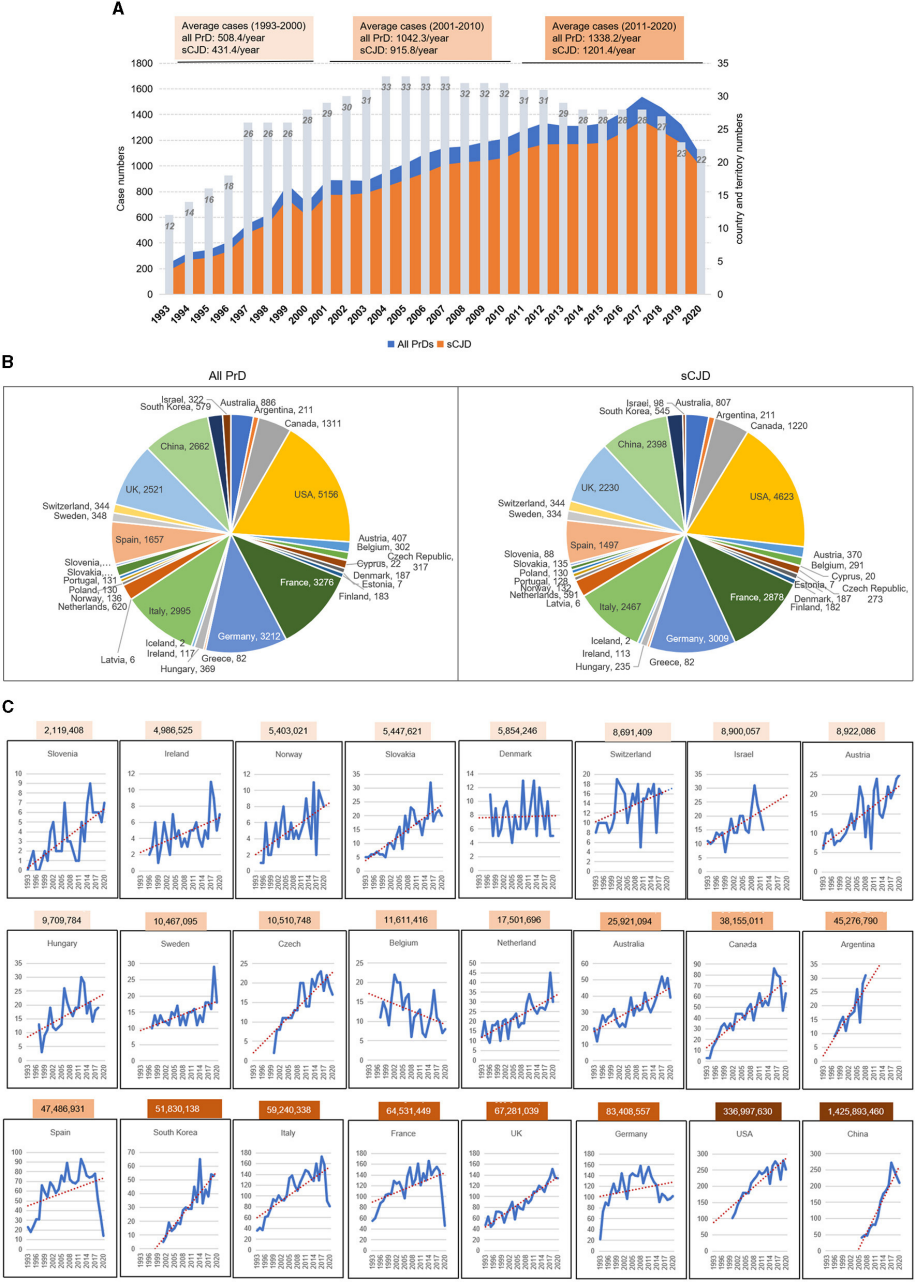
Spatiotemporal distribution of all PrD and sCJD cases worldwide from 1993 to 2020. **(A)** The annual reported case numbers of PrDs and sCJD in 34 countries with annual surveillance data. The gray columns represent the annual numbers of the countries with PrD data. The average and median case numbers in the periods 1993–2000, 2001–2010, and 2011–2020 are indicated above. **(B)** Distribution of the reported case numbers of all PrDs and sCJD in different countries. **(C)** The individual annual case number of PrDs in 24 countries from 1993 to 2020.

The case numbers of all PrD and sCJD from 32 countries with accessible surveillance data between 1993 and 2020 were collected. In total, 27,872 PrD cases and 24,623 sCJD cases were reported, of which sCJD cases occupied 88.3% of all PrDs. As shown in Figure 1A, the reported case numbers showed an increasing trend from 1993 to 2020. The average case numbers of all PrDs and sCJD per year were 508.4 and 431.4 in the initial 8 surveillance years (1993–2000), 1,042.3 and 915.8 in the second 10 years (2001–2010), 1,338.2 and 1,201.4 in the last 10 years (2011–2020), respectively.

Among the selected countries, Australia, Austria, France, Canada, Germany, Italy, the Netherlands, Slovakia, Slovenia, Spain, Switzerland, and the UK had continuous surveillance data from 1993 to 2020. Belgium, Denmark, Finland, Hungary, Ireland, Norway, Portugal, and Sweden had the data from 1996 or 1997 to 2020. Czech Republic, Korea, and China had the data from 2000, 2001, and 2006, respectively. Israel had the data from 1993 to 2012, while Green and Argentina had the data from 1997 to 2008. Due to the inaccessibility of the data from 2019 and 2020 for several countries, the PrD case numbers of PrDs and the reporting countries in the last 2 years were relatively fewer compared to those of 2016 and 2017 (Figure 1A).

The total reported case numbers of all PrDs and sCJDs in the selected countries are shown in Figure 1B. The countries with PrD case numbers over 1,000 were the USA (*n* = 5,156), France (*n* = 3,276), Germany (*n* = 3,212), Italy (*n* = 2,995), China (*n* = 2,662), the UK (*n* = 2,521), Spain (*n* = 1,657), and Canada (*n* = 1,311). The countries that reported more than 500 PrD cases were Australia (*n* = 886), the Netherlands (*n* = 620), and South Korea (*n* = 579). Eight other countries reported more than 300 cases, including Austria, Belgium, Czech Republic, Israel, Sweden, Switzerland, Slovakia, and Hungary. Furthermore, the annual diagnosed PrD cases in 24 countries were separately calculated. Except for Belgium, all countries showed increased tendencies for the annual diagnosed PrD cases in the surveillance years (Figure 1C). The case numbers varied largely among the countries but were generally associated positively with the national population sizes.

### Proportions of genetic PrD (gCJD, FFI, and GSS)

**Figure 2:**
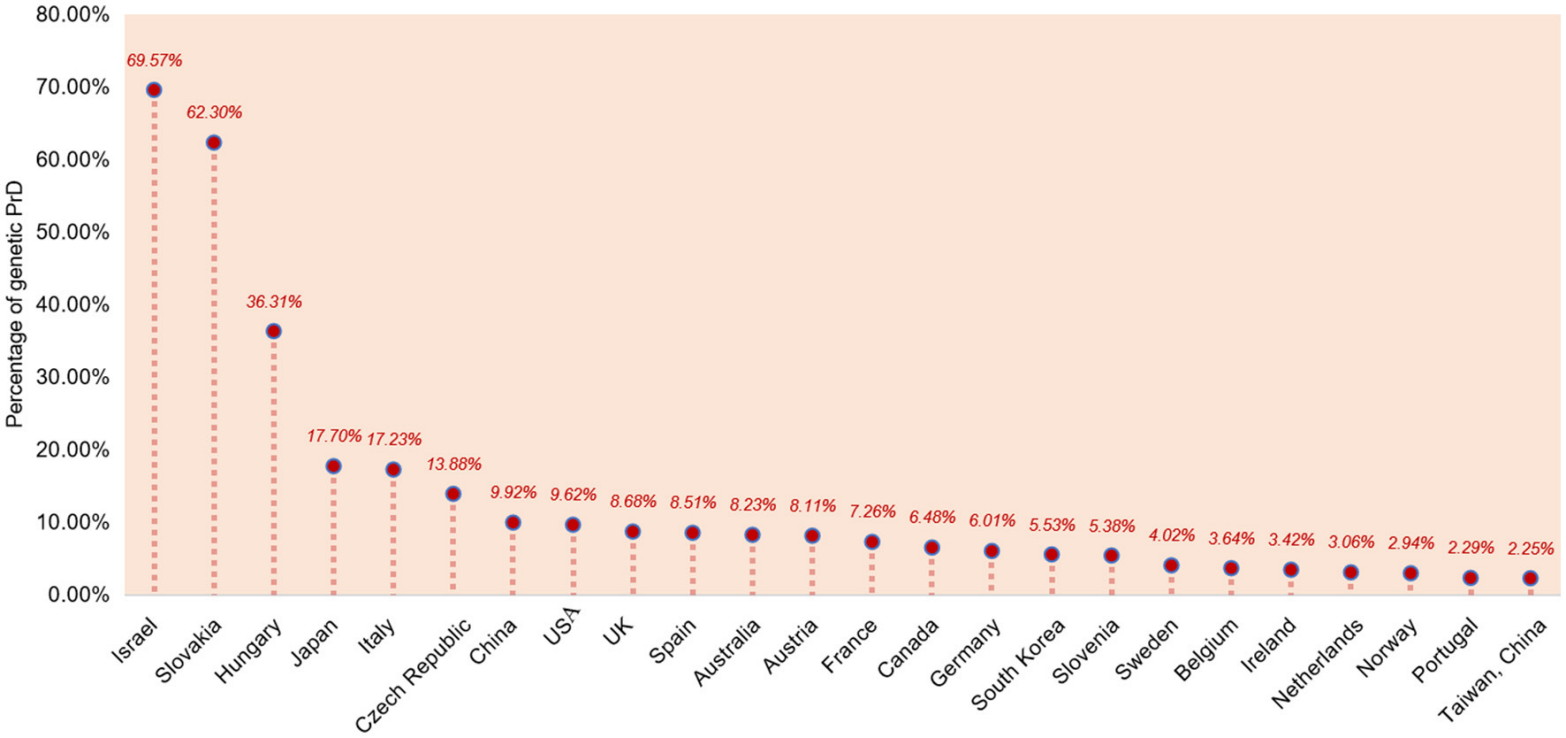
The percentages of genetic PrD out of all PrDs in 24 different countries and territories.

The case numbers of genetic PrD, including gCJD, FFI, and GSS, in dozens of countries were counted, and the percentages of genetic PrD out of all PrD were calculated (Figure 2). Among the 24 counties or territories, a total of 3,010 genetic PrD cases were reported according to the official websites and literature pieces (19, 20), which accounted for 10.83% of all reported PrD cases. The genetic PrD percentage of Japan was cited from a 10-year surveillance report (16, 21). Overall, the genetic PrD percentages of 11 countries were in the range of 5–10%, i.e., China, the USA, the UK, Spain, Australia, Austria, France, Canada, Germany, South Korea, and Slovenia. Seven countries or territories showed relatively low (below 5%) genetic PrD percentages, such as Sweden, Belgium, Ireland, the Netherlands, Norway, Portugal, and Taiwan-China. The genetic PrD percentages of Japan, Italy, and the Czech Republic were in the ranges of 10–20%. Israel, Slovakia, and Hungary had much higher genetic PrD percentages of 69.57, 60.30, and 36.31%, respectively.

### AMRs of sCJD worldwide

**Figure 3:**
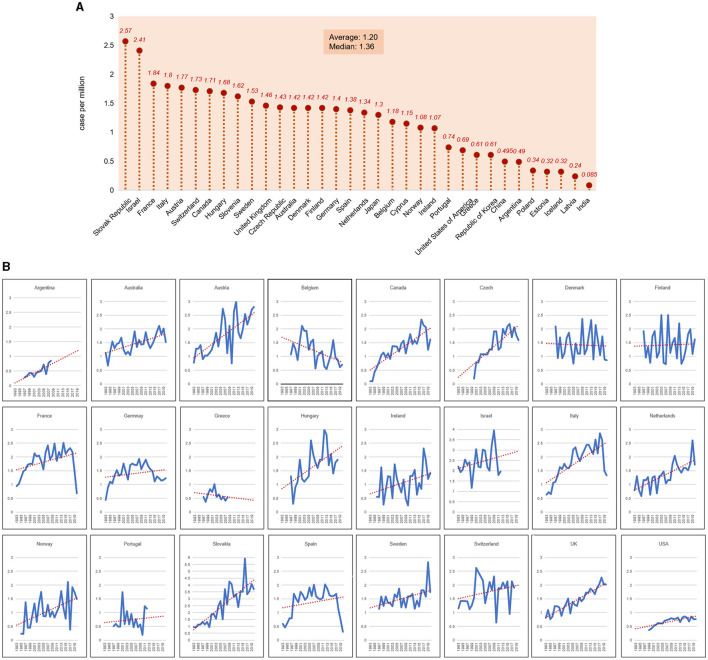
The annual mortality rates of sCJD worldwide from 1993 to 2020. **(A)** The national sCJD annual mortality rates of 34 countries after normalizing with the general population. The average and median annual mortality rates of sCJD among the countries are indicated in the graph. **(B)** The individual annual mortality rates of sCJD in 24 countries.

Based on the surveillance or reported case numbers of sCJD, the sCJD incidences in various countries were estimated. The average AMRs of sCJD from 1993 to 2020 are shown in Figure 3A. Among the 34 selected countries, Slovakia and Israel showed the highest AMRs, which were 2.57 and 2.41/million, respectively. The AMRs of 21 countries were higher than 1.0/million; among them, eight countries (France, Italy, Austria, Switzerland, Canada, Hungary, Slovenia, and Sweden) showed AMRs higher than 1.5/million. The alterations of the AMRs of 24 countries with consecutive surveillance data over 10 years from 1993 to 2020 were selected and are illustrated in Figure 3B. Most of the countries revealed an increased trend in the reported incidences.

### Distribution of acquired PrD (Kuru, vCJD, and iCJD) cases

To date, Kuru has only been detected in the Fore tribe in Papua New Guinea because of ritualistic mortuary cannibalism (22, 23). The Kuru epidemic peaked in the late 1950's (23) and subsided following the prohibition of cannibalism in the mid-1950's (24). However, Kuru did not manifest until several decades after exposure, due to the heterozygous mutation at codon 129 (23).

**Figure 4:**
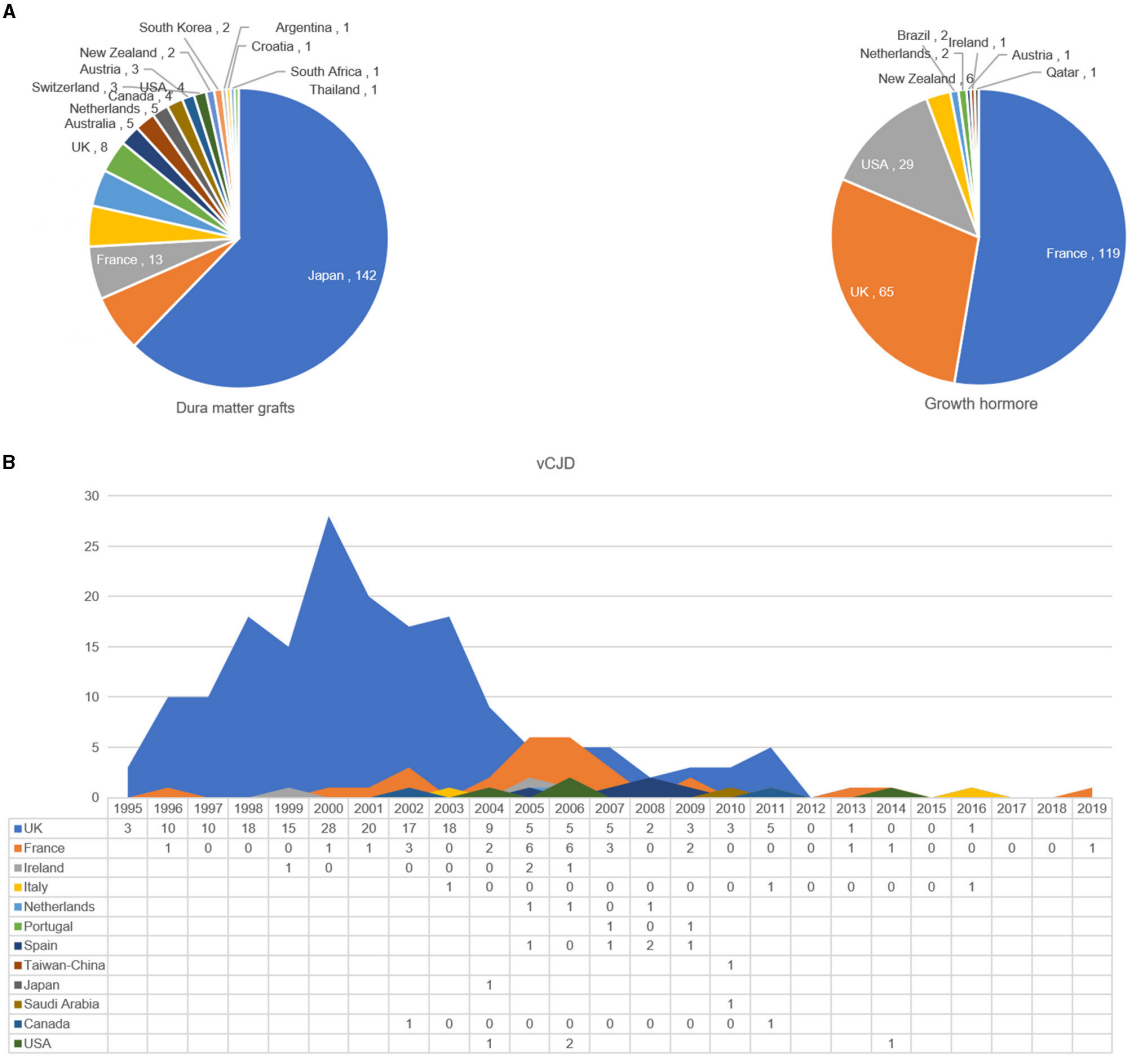
Distributions of iCJD and vCJD cases in different countries and territories. **(A)** Case numbers of iCJD caused by dura mater graft and growth hormone. **(B)** The distribution and annual case numbers of vCJD.

Another form of acquired prion disease is iCJD. There have been at least 485 iCJD cases reported worldwide; 96.7% of them were identified before 2012 (25). In addition to a small number of iCJD cases being caused by neurosurgical instrument contamination (four cases), contaminated electroencephalogram (EEG) needles (two cases), corneal grafts (two cases), and gonadotrophic hormone (four cases), the majority of the iCJD cases were caused by administrations of growth hormone (226 cases) and dura mater grafts (228 cases) derived from human cadavers with undiagnosed CJD contaminations (25). As shown in Figure 4A, 18 countries have reported iCJD cases associated with dura mater grafts; the top five countries were Japan (*n* = 142), Spain (*n* = 14), and France (*n* = 13). Nine countries have reported iCJD cases caused by growth hormones; the top three countries were France (*n* = 119), the UK (*n* = 65), and the USA (*n* = 29). Since 2013, there have been 16 iCJD cases reported: 11 in the UK, 4 in the USA, and 2 in Canada.

In total, 232 vCJD cases have been reported worldwide since 1995. The case numbers of vCJD reached its peak during the period from 1998 to 2003 and have decreased markedly since 2004 (Figure 4B). Globally, 12 countries or regions have reported definite and probable vCJD cases: 96.12% of vCJD cases in Europe, 2.59% in North America, and 1.29% in Asia. Overwhelmingly, more vCJD cases (*n* = 178) were identified in the UK, which accounted for 76.72% of all vCJD cases in the world, followed by France (12.07%, *n* = 28). Remarkably few cases were identified in the other countries or regions, including Spain (*n* = 5), the USA and Ireland (*n* = 4), Italy and the Netherlands (*n* = 3), Canada and Portugal (*n* = 2), Japan, Taiwan-China, and Saudi Arabia (*n* = 1). Moreover, the secondary infections with variant CJD transmitted by transfusion of blood products were verified in the UK (*n* = 3) in 2003 (26), 2004 (27), and 2006 (28). Compared to the period of the vCJD peak in the UK (1998–2003), the vCJD peak period for the remaining 11 countries or regions was late (2003–2008). Up to 28 cases were reported in the UK in 2000 and 21 in 2001 (Figure 4C).

### CJD cases reported in Africa

An early study described 23 CJD cases of North African immigrants to France (12 came from Tunisia and 11 from Algeria) from 1968 to 1982 (29). Another report briefly described 13 CJD cases (four definite, seven probable, and two possible sCJD cases) in Kenya from 1990 to 2004 based on a hospital (30). There were several case reports of CJD in African countries, e.g., the first sCJD case in Morocco in 2005, which was confirmed by postmortem histology (31), a probable CJD in Egypt in 2019 (32), and the first Heidenhain variant of sCJD from East Africa in 2021 (33). In 2006, South Africa reported the first dura mater graft-associated iCJD cases (34). A few gCJD cases, such as V201I in a Moroccan patient and a 5-octapeptide repeat insertion (5-OPRI) in a South African family, were reported (35, 36).

## Discussion

In this present study, we have reviewed the accessible surveillance data of PrD cases from more than 30 countries worldwide from 1993 to 2020. The majority of the surveillance data comes from countries in Europe, North America, and Eastern Asia. Although CJD has been described for a long time, the global surveillance for CJD/PrD has been implemented since the 1990's under the framework of the WHO, along with the emergence of BSE in cattle and vCJD in human (2). The EuroCJD program started in 1993 and consisted of seven countries (Austria, France, Germany, Italy, the Netherlands, Slovakia, and the UK) initially and expanded to other European (Spain) and non-European (Australia and Canada) countries later. In 2007, another broader surveillance program, NeuroCJD, was initiated, covering almost all European countries. Meanwhile, Argentina, Australia, Japan, Canada, Mexico, China, Israel, and the USA, among other countries, conducted their national surveillance programs and joined the global network.

The annual identified PrD case numbers and PrD-reported mortality, either worldwide or in the country or territory, increased from 1993 to 2020, with there being an almost 2-fold increase. One of the most important reasons for such increases is the implementation of ~30 years-long surveillance for CJD globally, which remarkably improves the awareness of PrD both in the professional field and the public community. Along with the progression of the overall diagnosis, the development and progression of new and specific techniques help greatly to recognize and diagnose the PrD cases, such as brain MRI, RT-QuIC, and the detection tools for some cerebral spinal fluid (CSF) proteins. The new techniques have led to a revision of the WHO-accepted diagnostic criteria on more than one occasion, so case classification has altered, with some cases (previously in possible or doubtful categories) currently being reported as probable. Additionally, the aging of the population worldwide in the past three decades may also influence the PrD annual incidence.

As a rare neurological disease, the recognition and diagnosis of PrD/CJD are still difficult in many low- and lower-middle-income countries. A few CJD cases have been reported in the literature in some African countries. In Asia, only Japan, South Korea, Mailand-China, and Taiwan-China conducted long-term PrD surveillance programs. A total of about 30 CJD cases have been reported in India over the past 30 years (37); since then, only a few studies have reported CJD (38–40). Sporadic cases of PrD have been reported in several other Asian countries, such as Pakistan, Thailand, Omen, and Singapore (41–43). A few sCJD cases have been reported in Malaysia (44). Among the countries of Middle and South America, some such as Argentina, Chile, Mexico, Brazil, and Peru have reported their data of PrD surveillance or studies. For example, in Argentina, 211 cases have been reported from 1996 to 2007; in Chile, 230 cases from 2001 to 2007 (45); in Mexico, 29 cases from seven individual studies from 1990 to 2020 (46) and 24 cases in a referral center during 2014–2019 (47); in Brazil, 35 cases during the period 2005–2007 (48), 408 sCJD cases, and several gCJD cases during the period 2005–2020 (48); and in Peru, 11 and six sCJD cases and several gCJD cases from various literatures (49, 50). It is apparent that the PrD or CJD case numbers globally are markedly undervalued, which may pose a potential biosafety risk.

The ratios of genetic PrD cases, including gCJD, FFI, and GSS, in the large portions of the counties recruited in this study are in the expected range (5–15%). Israel and Slovakia show extremely high ratios (over 60%) of genetic PrD. Early studies have already addressed the fact that the incidence of a kind of gCJD, E200K, is frequent among Jews of Libyan origin, which was estimated to be 100 times higher than that in the general population (51–53). Recently, E200K gCJD patients of Turkish ancestry were analyzed, and it was found to display similar demographic and clinical features to those of Libyan descent (54). E200K gCJD is also frequent in Slovakia. A previous study identified the familial clusters among 78 definite CJD cases in Slovakia and an adjacent part of Hungary from 1972 to 1991 (55). Another study has shown that 74.2% (95/136) of Slovakian CJD cases contain the E200K mutation (56). Surveillance of PrD cases in Eastern Slovakia from 2004 to 2016 has identified 21 E200K gCJD and six sCJD cases, with E200K gCJD accounting for 77.78% of the total number of cases (57). The ratio of genetic PrD in Hungary is also much higher (36.35%). Similar to the neighboring country of Slovakia, E200K gCJD is the predominant subtype, which is presumed to relate to the historical migration of the Slovakian population or to being geographically close to Slovakia (58). Japan and Italy also show relatively high ratios of genetic PrD, ~17–18%. More frequent CJD cases with the V180I variant in Japanese (59) and the V210I variant in Italian (60) are possibly associated with those phenomena. On the other hand, the ratios of genetic PrD are very low in several European countries, such as Belgium, the Netherlands, and the Scandinavian countries, such as, Switzerland and Poland, where even fewer genetic PrD cases are reported. In addition to the accessibility of *PRNP* sequencing, the ethnic-associated distribution of genetic PrD and its subtypes among various countries is apparent.

Acquired CJD has dropped dramatically in the recent decades worldwide. Kuru has been almost eradicated in Papua New Guinea after the prohibition of cannibalism. The two most predominant forms of iCJD, administrations of growth hormone and dura mater grafts, have diminished significantly after the prohibition of their use. It is noteworthy the number of vCJD cases both in the UK and globally has been almost undetected in the past 3 years after the successful implementation of prevention and control measures to remove BSE prions from the animal and human food chains. It is clear that the removal of prion sources successfully contains and eliminates the occurrence and outbreak of human-acquired PrD.

The main surveillance data in this study are derived from the CJD international surveillance network supported by the European Union (EU) CDC and some accessible national CJD surveillance websites. Some countries have only the total numbers of various subtypes of PrDs in a certain period, but without the exact annual numbers. Japan is one of the countries implementing national CJD surveillance programs worldwide. However, the precise annual numbers of PrDs, particularly the data from the last 10 years, are inaccessible. Additionally, the PrD annual data from Chile, Brazil, and Mexico are also lacking. Hence, the global annual PrD number in this context is significantly underestimated. Given the annual mortality of all PrDs of 1 case/million and the global total population of 8.032 billion in 2023 (61), the PrD annual numbers are estimated to be at more than 8,000 at least. Only less than one-fifth of the PrD cases can be recognized and handled properly worldwide, and more importantly, most diagnosed PrD cases are distributed in high- and upper-middle-income countries or territories. As a transmissible fatal neurodegenerative disease lacking prophylactic and therapeutic tools, active international PrD surveillance for both humans and animals still remains vital to eliminate the threat of prion disease from a public health perspective (62).

## Conclusion

In the present study, an epidemiological retrospective analysis of PrD was conducted with the purpose of better understanding the spatiotemporal distribution of features across the globe from 1993 to 2020. The case numbers and annual incidence of all types of PrDs reveal an increasing trend, while those of vCJD and iCJD declined remarkably. The surveillance programs are still limited to high- and upper-middle-income countries or territories in Europe, North America, East Asia, and Oceania. A lack of awareness, poor clinical and laboratory capacity, and limited financial resources prohibit the comprehensive understanding of human PrD trends globally. The potential threats of both human and animal prions are far from eliminated. Thereby, the integration and expansion of human and animal PrD monitoring networks in all regions of the world are still needed and should be improved, including the development of simplified and user-friendly detection technologies, digitalized information collection and analysis systems, and easily accessible tissue banks.

## Data availability statement

Publicly available datasets were analyzed in this study. This data can be found at: https://www.eurocjd.ed.ac.uk/data_tables.

## Author contributions

L-PG: Data curation, Methodology, Software, Writing – original draft. T-TT: Data curation, Formal analysis, Methodology, Software, Writing – review & editing. KX: Software, Writing – review & editing. CC: Methodology, Writing – review & editing. WZ: Methodology, Validation, Writing – review & editing. D-LL: Data curation, Formal analysis, Writing – review & editing. R-DC: Formal analysis, Investigation, Writing – review & editing. QS: Supervision, Writing – review & editing. X-PD: Writing – original draft.
